# Quality evaluation of online platforms information for retail of prescription medicines in China: an observational study of the case of paroxetine

**DOI:** 10.1186/s12911-026-03386-4

**Published:** 2026-02-21

**Authors:** Han Yao, Chutong Li, Xiaonan Shi, Bo Peng, Zhenyang Kang, Jing Sun, Yuanli Liu

**Affiliations:** 1 School of Health Policy and Management, Chinese Academy of Medical Sciences & Peking Union Medical College, Beijing, 100730 China; 2 School of Population Medicine and Public Health, Chinese Academy of Medical Sciences & Peking Union Medical College, Beijing, 100730 China; 3 National Research Institute for Family Planning, Beijing, 100081 China; 4 Graduate School of Peking Union Medical College, Beijing, 100730 China

**Keywords:** Quality, Information, E-commerce, Platform, Online, Retail, Prescription medicines, Paroxetine hydrochloride, DISCERN

## Abstract

**Objectives:**

This study aimed to generate evidence to help improve the quality of information provided by Chinese online platforms for retail of prescription medicines, as a baseline for enhancing the supervision by the regulatory authorities of internet retail of prescription medicines to offer comprehensive, accurate and reliable information to consumers.

**Methods:**

This is an observational study to investigate the quality of the information provided by two key types of online platforms in China that sell paroxetine online, a common mental health medication. The authors utilized the DISCERN with the Likert 5 scale, a validated instrument comprising three domains and 16 items to assess the comprehensiveness, accuracy and reliability of information provided by a total of 34 Chinese online platforms. We firstly examined whether these online platforms have been diligent in their duty to provide appropriate retail service-related information (e.g. delivery, payment, return policies) for prescription medications. We also evaluated the quality of the medication information associated with treatment plans for specific prescriptions provided by the online platforms. Three trained master medical students independently conducted the evaluation. By securing the consistency of the scoring results among different evaluators and the reliability of the evaluation, we adopted the mean score of the three evaluators for each item to perform further analysis at the item and domain levels, and between two types of online platforms.

**Results:**

Among the 34 online platforms, 2 were evaluated as providing excellent information for retail of paroxetine (1/34, 5.88%), 6 were good (6/34, 17.65%), 25 were fair (25/34, 73.53%), and 1 was poor (1/34, 2.94%). The key identified flaws of the online platforms for providing appropriate information for retail of paroxetine included (1) inadequate provision of medication information, (2) absence of prescription validation and authentication, and (3) lack of professional pharmacy services such as mandatory pharmacist consultations or patient-specific drug counselling.

**Conclusions:**

Consumers face challenges in obtaining comprehensive, accurate and reliable information from the online platforms for common mental health prescription medications. The quality of information provided by the online platforms for retail of prescription medicines is generally suboptimal, and the compliance of prescription validation and authentication is problematic. These highlight the need for strengthening the supervision of the online platforms with novel approaches along with their rapid developments, and ensuring appropriate online information provided to consumers.

**Supplementary information:**

The online version contains supplementary material available at 10.1186/s12911-026-03386-4.

## Introduction

Online pharmaceutical retail has been expanding quickly with new developments in internet technology [1]. More recently, the COVID-19 pandemic has profoundly impacted individual lifestyles and healthcare-seeking behaviours. These changes increased the demand for internet retail of medicines, including some specialty prescription medications [2, 3]. Information to patients played an important role in treatment, and treatment compliance increased with more effective information provided to patients [4]. Although online retail of medicines is convenient for consumers, there are also risks associated with poor quality of information provided online, in terms of comprehensiveness, accuracy and reliability. Biased information provided online may create an illusion of reliability, and mislead consumers [5]. There has been a concern that internet pharmacies sell prescription medicines without a valid physician order [6]. Furthermore, the sale of falsified or substandard medicines through online platforms poses significant safety risks to consumers. A report relying on industry data from 2016 disclosed that 96% of online pharmacies worldwide failed to adhere to applicable legal requirements, and 92% of illegal practices involved selling medicines without valid prescriptions [7].

China first allowed online retail of prescription medications in 2005. This policy was discontinued later due to patient safety concerns but eventually restarted in 2022, with the promulgation of the Provisions for Supervision and Administration of Pharmaceutical Online Sales [8]. Thus, China established a set of regulatory rules for online retail of prescription medications. In the early development stage, pharmaceutical enterprises built in-house transaction functions on their websites, which evolved along with the cooperation with third-party e-commerce platforms. Gradually, e-commerce enterprises that were not originally active in pharmaceutical sales also involved in online retail of medicines, like JD.com [9]. At present, online and offline pharmaceutical enterprises run retail businesses either on the third-party online platforms or build their own online pharmacies [10]. There are mainly four types of online platforms now operating in China, including Business to Business (B2B), Business to Customer (B2C), Customer to Customer (C2C), and Online to Offline (O2O). B2B facilitates transactions between wholesalers and manufacturers for the distribution of pharmaceuticals to a wide network of retailers and healthcare providers. B2C and O2O are the two main types of online platforms that provide retail service for prescription medications via the internet. B2C represents the Business to Customer model, under this model, pharmaceutical companies (including manufacturers, wholesalers and retailers) sell prescription medicines directly to consumers, either via their own flagship online stores or by other pharmaceutical enterprises via third-party e-commerce platforms (act as the intermediaries to offer a broad range of products). O2O is an Online to Offline model run by the pharmaceutical retail enterprises to run internet pharmacies with remote transactions. Under this model, online orders are filled by offline physical pharmacy retail stores operated by individual pharmaceutical retail enterprises. O2O is typically branded under the names of the retail pharmacies. They often provide additional services such as online consultations, prescription refills, and home delivery, integrating the online order process with offline pharmacy store operations. In 2021, 918 companies were granted with the Internet Medicines Trading Service Qualification Certificate by the Chinese medicines regulatory authorities [11]. The pharmaceutical e-commerce enterprises in China reached USD 30 billion, accounting for 8.3% of total sales of the overall pharmaceutical market [12]. In the first half of 2022, the sales of prescription medications through B2C and O2O online platforms offered retail services accounted for about 25 and 35% of the total sales, respectively [13, 14], showing ample room for development.

The acceptance of online retail of prescription medications in China represents a trend in socioeconomic development. However, this requires close monitoring, tight supervision with novel strategies to keep pace with the rapid developments of the internet market. By searching the databases of the China National Knowledge Infrastructure and PubMed from their inception until March 8, 2023, with the keywords ‘online prescription drug/medicines’, ‘medication safety’, and ‘China’, we found a series of critical problems of online pharmacies. The main problems include trading fake and substandard products [15, 16], fake prescriptions [17], exaggerated efficacy [18], consumer privacy leakage [19], and regulatory failure [20]. We did not find any studies that demonstrated improved regulatory compliance after the introduction of the Provisions for Supervision and Administration of Pharmaceutical Online Sales in August 2022.

According to the report of the World Health Organization (WHO), depression is a common disease and one of the important factors in the overall burden of global diseases [21]. Meanwhile, depression and anxiety are also the top two major causes of disability and suicide [22], which have a huge impact on personal, family, and social mental health. According to the briefing released by the WHO in March 2022, COVID-19 has led to an increase of about 25% in the prevalence of anxiety and depression worldwide [23]. In China, there are about 95 million patients with depression [24], and anxiety disorder is the most common type of lifelong disorder [25]. According to the survey of the 2022 National Blue Paper of Depression [24], 92% of patients accepted online consultation from a telehealth provider, 27% accessed medicines online, 47.7% did not have enough medication information about the medicines they used, and only 9.9% of patients knew drug reduction methods. Patients with depression are also worried about how to avoid adverse reactions at a safe and effective dose of medication. Notably, the population with depression and anxiety in China tends to get younger [24]. Teenagers and adolescents are more likely to seek health and medication advice through an online search and purchase medicines, including many prescription medications from online platforms [26].

Paroxetine, with the ATC code of N06AB [27], is a selective serotonin reuptake inhibitor, that should be purchased with a prescription from a health professional. Good tolerability and efficacy have made this medicine popular [28]. According to the ‘Clinical Practice Guide for the Treatment of Depression Across Three Age Cohorts’ released by the American Psychological Association, paroxetine is the first choice for persistent depression, panic disorder, and social phobia [29]; it is also a first-line antidepressant in China [30]. However, due to its pharmacological properties and dangerous withdrawal symptoms, it may increase the risk of suicide in children and adolescents. Anticholinergic side effects and contraindications in older adults also make it controversial and require special attention to ensure medication safety [29]. This study aimed to fill this gap by assessing the quality of information provided by the online platforms for retail of prescription medicines in China. Therefore, paroxetine served as a suitable tracer drug for this evaluation due to its common use, specific safety profile including withdrawal risks, age-related contraindications, and the consequent critical need for comprehensive and accurate patient information.

## Methods

### Study design

This is an observational study which assessed the comprehensiveness, accuracy and reliability of information provided by two types of online platforms in China that run retail services of prescription medications. We took paroxetine, a common prescription medicine for mental health problems, to conduct a case study. Trained investigators utilized the DISCERN, a standardized survey tool to gather both qualitative and quantitative evaluation data. By employing content analysis, we systematically evaluated the quality and utility of the text, images, and other forms of contents presented on the online platforms for consumers to purchase prescription medications online. The platform assessments were conducted between October and November 2022.

### Population and sample

**Fig. 1:**
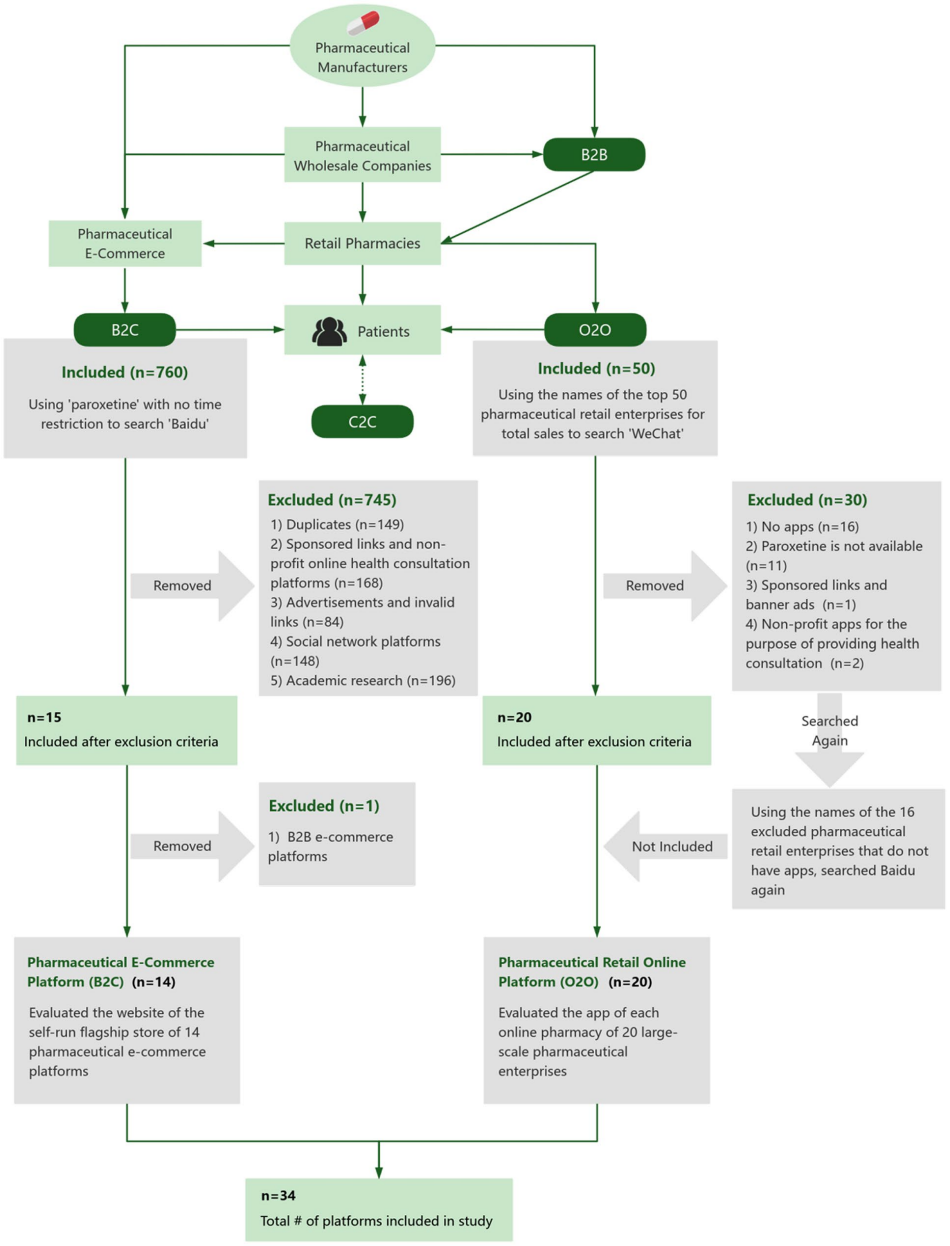
Business model of online platforms and a flowchart for selection of online platforms that provide retail services of prescription medications. Notes: B2B = Business to Business, refers to online platforms facilitating transactions between businesses; B2C = Business to Customer, indicates e-commerce platforms where businesses sell directly to consumers; C2C = Customer to Customer, involves peer-to-peer transactions; O2O = online to offline represents platforms that bridge online interactions with offline services, typically through applications; app = application

We targeted two key types of online platforms ‘pharmaceutical e-commerce platforms’ (B2C) and ‘pharmaceutical retail online platform (O2O)’ [31]. In this manuscript, ‘online platforms’ serves as the umbrella term for both types, while the specific designations B2C and O2O are used when a distinction between the two models is necessary. Details of these online platforms, as well as the search and inclusion procedures were presented in Fig. 1. In brief, we simulated consumer searches to identify platforms retailing paroxetine, which involved a broad initial search followed by a structured screening process against predefined eligibility criteria. The detailed procedures for each platform type are described in the following subsections.

#### Pharmaceutical e-commerce platform (B2C)

Baidu is one of China’s internet giants. By December 2022, the market share of Baidu accounted for about 65% of China’s search engines [32]. We adopted the consumers’ perspectives to search Baidu using the keyword ‘paroxetine’ with no time restriction to identify any online platform that provides retail service of paroxetine. We excluded B2B and C2C e-commerce platforms, duplicates, sponsored links, and non-profit online health consultation platforms, advertisements, invalid links, social network platforms, and academic research. Considering that internet pharmacies that operate on the same B2C pharmaceutical e-commerce platform adopt the same structure of webpage display and purchasing procedure, only all the self-run flagship stores of the 14 B2C pharmaceutical e-commerce platforms were included for evaluation.

#### Pharmaceutical retail online platform (O2O)

Pharmaceutical enterprises run their own online business via either self-built smartphone applications (apps) to provide more conducive online service with better interactions with the users, or set up internet pharmacies on the pharmaceutical e-commerce platforms [33]. Small-scale pharmaceutical retail enterprises rely only on pharmaceutical e-commerce platforms to run online retail businesses. Considering that each pharmaceutical e-commerce platform adopts a uniform structure of webpage display and purchasing procedure for all the merchants, which have been included in the analysis of B2C, we searched for the apps of large-scale pharmaceutical retail enterprises only. As businesses operated on apps could not be identifiable from the website, we used the app search function in WeChat (a multifunctional social media, messaging, and mobile payment app widely used in China) with the names of the large-scale pharmaceutical retail enterprises one by one. For those that were confirmed to have apps, the evaluators opened the apps and searched with the keyword ‘paroxetine’. The inclusion criteria were as follows, (1) apps of pharmaceutical retail enterprises listed in the top 50 for total sales per the Chinese Ministry of Commerce in 2021 and (2) apps that run an online retail business of paroxetine. The exclusion criteria included, (1) pharmaceutical retail enterprises listed in the top 50 for total sales per the Chinese Ministry of Commerce in 2021 that had no apps; (2) apps of sponsored links and advertisements; and (3) apps of non-profit online health consultation platforms. We used the names of the 16 excluded pharmaceutical retailers to search in Baidu and found none of their online retail businesses on the website. 20 internet pharmacies owned by large-scale pharmaceutical retail enterprises were include for evaluation. All 34 target online platforms were listed in Appendix 1. Among the target 34 online platforms, each provided unique information on the homepages of their websites for retail of paroxetine.

### Evaluation tool

There are several tools to evaluate the quality of health information, including HONcode (created by Health on the Net), Michigan Checklist (created by the University of Michigan), LIDA (created by Minervation Consulting, UK), and DISCERN (developed by the University of Oxford and funded by both the British Library and National Health Service). The first three tools provide an expert’s perspective, while DISCERN provides a user’s perspective [34]. Since most consumers are not experts but users, we adopted DISCERN with the five-point Likert scale to perform the evaluation. Although previous studies used DISCERN to evaluate the quality of health information provided by the websites that provide knowledge concerning diseases such as diabetes, psoriasis, bronchial asthma, and breast cancer [35–38], no one used it to evaluate the quality of the information provided by the online platforms for retail of prescription medications. In recent years, relevant studies have used the DISCERN to evaluate the reliability of information about periodontal disease provided to patients by ChatGPT-4 [39], as well as the credibility of YouTube online videos as an educational source for patients with cervical spondylosis [40]. These studies observed good reliability and validity of the DISCERN. This study utilized the Chinese version of DISCERN with validated internal consistency and inter-rater reliability, which ensured consistent results obtained from different evaluators [41]. As presented in the Appendix 2, the DISCERN consists of three key domains. Domain 1 evaluates the comprehensiveness and reliability of the information presented on the homepages of the websites of the online platforms. Domain 2 evaluates the accuracy of information about treatment options. Domain 3 consists of a single overall quality rating item. It allows the evaluator to synthesize judgments from Domains 1 and 2 into a holistic score reflecting the perceived overall utility and reliability of the platform’s information for supporting treatment decisions.

### Measurement

We evaluated the comprehensiveness, accuracy and reliability of the information provided by the online platforms for retail of paroxetine in three domains with 16 items. For each item, we used a five-point Likert scale, where 1 indicated strong disagreement and 5 indicated strong agreement with the statement. The first domain included eight items about the information presented on the homepage of the website for retail of prescription medicines, which is not directly associated with medicines or treatments. The second domain included seven items about the medication information provided by the online platforms related to a particular treatment scheme (in this study, involving depression, anxiety, and paroxetine hydrochloride). The third domain included one item about the overall quality of the information provided by the online platforms in terms of comprehensiveness, accuracy and reliability [42]. We developed detailed evaluation criteria for each item based on expert consultations, literature review, the Provisions for Supervision and Administration of Pharmaceutical Online Sales, and the manufacturer instructions for paroxetine approved by the Chinese medicines regulatory authority [43, 44]. The criteria for evaluating the presence and adequacy of professional pharmacy services (e.g., items relating to support for decision-making) were informed by key requirements outlined in the Provisions for Supervision and Administration of Pharmaceutical Online Sales [8, 43], which emphasize the role of licensed pharmacists in providing online consultation services and prescription review. Details were presented in Appendix 2.

### Evaluation strategy

Three master’s medical students were trained to have uniform understanding of each item of the DISCERN and its application, and independently performed a parallel scoring of the 34 online platforms in random order. To standardise the evaluation criteria and to ensure inter-evaluator consistency, the evaluators firstly conducted the pre-tests on three B2C online platforms and three O2O online platforms. When there were different evaluation opinions, the evaluators jointly discussed the evaluation criteria, the pre-tests continued until they reached an agreement and the scoring results among the evaluators were consistent [45]. The average scoring result for each item was deemed credible for further analysis.

### Statistical analysis

We constructed the dataset with Excel 2019 (Microsoft Corporation, Redmond, WA), and used IBM SPSS 26.0, R 4.1.2, and Prism 9.0.0 (GraphPad Software Inc, San Diego, CA) to conduct the statistical analyses. We calculated the $${r_{WG}} $$ value to conduct a within-group agreement test, which was used to gauge the consistency level between the scoring results submitted by the three evaluators. According to the empirical criteria, consistency is sufficient at $${\rm{ }}{{\rm{r}}_{{\rm{WG}}}} > 0.6$$ or 0.7 [46, 47]. We set 0.7 as the ideal level, which meant when $${\rm{ }}{{\rm{r}}_{{\rm{WG}}}} > 0.7$$, we considered that the variation among different evaluators was acceptable, and it was reasonable to aggregate all three scoring results for the same online platform. We calculated $${\rm{ }}{{\rm{r}}_{{\rm{WG }}}}$$ as follows:

3.1 $${r_{WG}}\left( j \right) = {{J\left[ {1 - \left( {{{\overline {S_{xy}^2} } \over {\sigma _{eu}^2}}} \right)} \right]} \over {J\left[ {1 - \left( {{{\overline {S_{xy}^2} } \over {\sigma _{eu}^2}}} \right)} \right] + \left( {{{\overline {S_{xy}^2} } \over {\sigma _{eu}^2}}} \right)}}.$$

${\rm{ }}J{\rm{ }}$ is the number of scale items, $S_{xy }^2$ is the variance of the scoring results, $\overline {S_{xy }^2}$ is the mean of the variance of the scoring results for multiple items, and $\sigma _{eu }^2$ is the expected variance of the hypothesised distribution.

The reliability of the scoring results depended on not only the within-group agreement of scoring results across online platforms but also the within-group variance between evaluators. Some scholars have argued that $$ {r_{WG}}$$ may not reflect inter-group variance, while others have suggested that inter-group variance should also be calculated as a necessary condition for arguing the validity of the overall structure [48]. We used the intraclass correlation coefficient (ICC) to test within-group reliability. Specifically, we tested the relative consistency of the scoring results from all three evaluators across the 34 online platforms using $${\rm{ICC }}\left( 1 \right)$$ and $${\rm{ICC }}\left( 2 \right)$$, as follows:

3.2 $$ICC\left( 1 \right) = {{MSB - MSW} \over {MSB + \left[ {\left( {k - 1} \right)MSW} \right]}}, $$

3.3 $$ICC\left( 2 \right) = {{MSB - MSW} \over {MSB}}.$$

Here, $k{\rm{ }}$ is the number of scoring results for each question item, *MSB* is the mean square between groups, and *MSW* is the mean square within groups. ${\rm{ICC }}\left( 1 \right)$ compares the relative magnitude of inter-group variation to within-group variation among the 34 online platforms, reflecting inter-group variability. ${\rm{ICC }}\left( 2 \right)$ is the average of the group mean reliabilities of the 34 online platforms, which reflects the reliability of the mean of all scoring results [49]. Empirical criteria consider ${\rm{ICC }}\left( 1 \right)$ greater than 0.21 and ${\rm{ICC }}\left( 2 \right)$ greater than 0.5 as ‘acceptable’ [50, 51]. This study adopted the ideal levels of ${\rm{ICC }}\left( 1 \right) > 0.21$ and ${\rm{ICC }}\left( 2 \right) > 0.66$, which were the mean levels of 189 studies from 1998 to 2012, as reported by Woehr et al. [52, 53].

By using the mean scoring result of the three evaluators as the result of each question, the sum of the mean scoring results for items Q1 to Q16 was regarded as the overall score of each online platform:

3.4 $$Sum = \mathop \sum \limits_{i = 1}^{16} {Q_i}.$$

We also calculated the scores for the three domains by summing up the mean scores for items Q1 to Q8, Q9 to Q15, and Q16, respectively.

Finally, we evaluated the quality of the information provided by the online platforms according to the scoring results. Good quality for Domain 1 meant that, the required information were presented on the homepage of the website of the online platform for retail of prescription medicines as required by the regulatory authority; good quality for Domain 2 implied that, the online platforms provide a full spectrum of accurate information with transparent and reliable sources, about the sold prescription medicines, including indications, contraindications, side effects, and interactions, which should align with the latest expert consensus; good quality for Domain 3 indicated an overall user-friendly online retail service experience which enabled the consumers to make well-informed medication decisions with controlled risks. A platform considered to be *Excellent* must have at least two domains rated as excellent. A *Good* rating was given when two or more domains rated as good, and the domain not rated lower than fair. If two or more domains were rated as poor, the platform was deemed *as Poor*. All other platforms that do not meet the criteria for *Excellent, Good,* or *Poor* were categorized as *Fair*. We then tested for differences in scoring results between the two online platform types (*p* < 0.05 considered significant$,{\rm{ \alpha }} = 0.05$).

## Results

We analysed a total of 34 online platforms, including 14 pharmaceutical e-commerce platforms and 20 pharmaceutical retail online platforms. The within-group agreement of the scores for each online platform was calculated with Eqs. 3.1. The inter-evaluator reliability was high. The mean within-group agreement test value (${r_{WG}}$) for all 34 online platforms was 0.951(*SD* = 0.066, range: 0.715–0.997), exceeding the 0.70 threshold. The intraclass correlation coefficients also indicated excellent reliability(${\rm{ICC }}\left( 1 \right) = 0.764$, ${\rm{ICC }}\left( 2 \right) = 0.906$; *F* = 10.689, *p* < 0.001). These results confirm that the scoring system was robust and that the averaged scores were suitable for subsequent analysis.

### Scoring results and attribute distribution

**Fig. 2:**
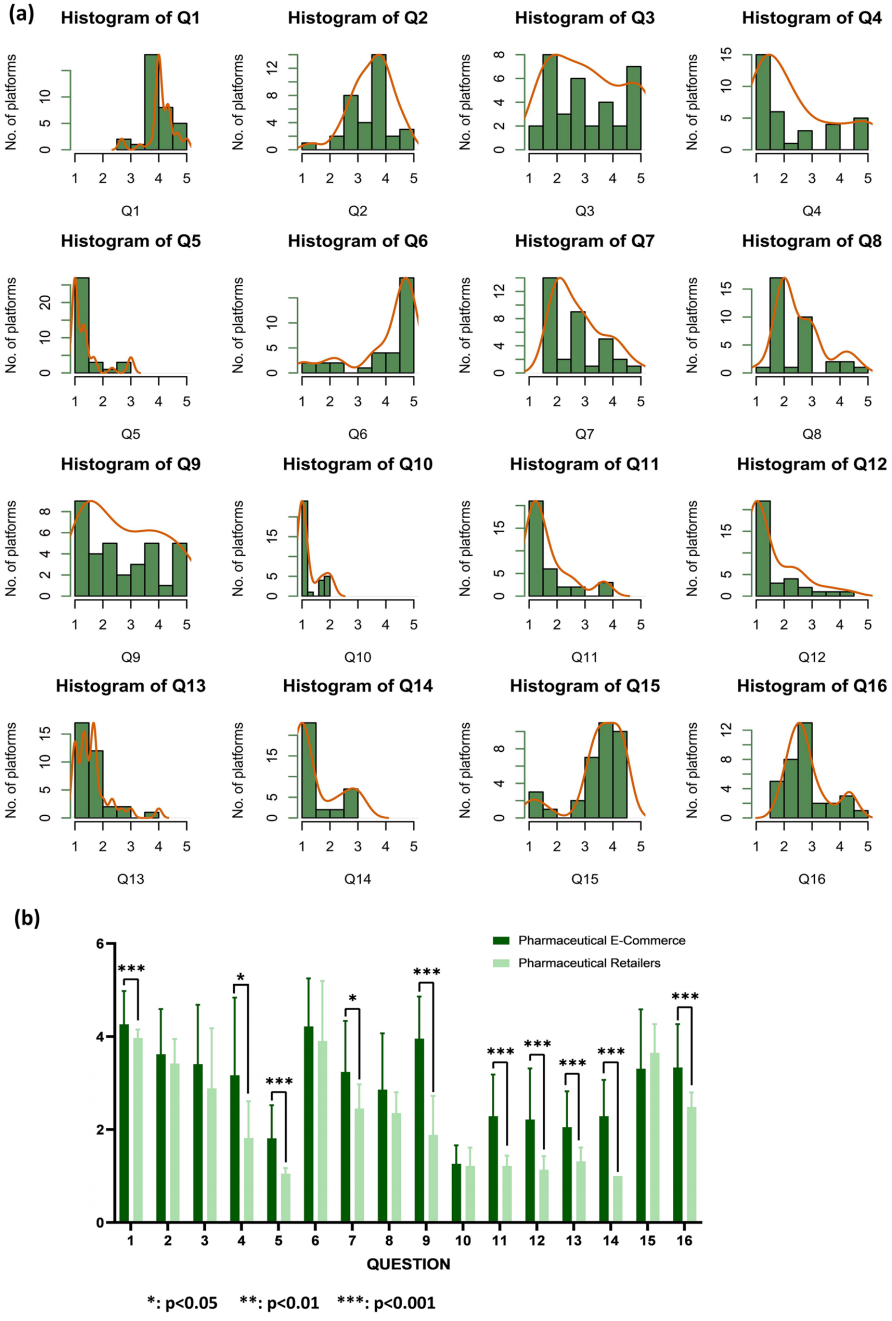
(**a**) Distribution of scoring results for each item of question across all 34 online platforms; (**b**) Differences of Likert scoring results of each DISCERN item of question between pharmaceutical e-commerce (B2C, *n* = 14) and pharmaceutical retail online (O2O, *n* = 20) platforms

Table 1 showed the mean Likert scores for each item across the 34 online platforms. Figure 2 (a) presented the distributions of the Likert scores of each item. The distributions of 12 out of 34 online platforms skewed left. This indicated that for a majority of platforms, scores clustered toward the lower end of the scale for these items, highlighting common deficiencies.

**Table 1:** Overall DISCERN scoring results for all target 34 online platforms

Domain		Questions	Mean (SD)	Min	Max
**Domain 1** Quality of information presented on the homepage of the website for retail of prescription medicines, not directly associated with medicines	**Q1—Q8**		**23.79 (4.31)**	**15.66**	**33.34**
	Q1	Are the aims clear?	4.09 (0.49)	2.67	5
	Q2	Does it achieve its aims?	3.50 (0.74)	1.33	4.67
	Q3	Is it relevant?	3.10 (1.29)	1.33	5
	Q4	Is it clear what sources of information were used to compile the publication (other than the author or producer)?	2.37 (1.38)	1	5
	Q5	Is it clear when the information used or reported in the publication was produced?	1.36 (0.59)	1	3
	Q6	Is it balanced and unbiased?	4.03 (1.19)	1	5
	Q7	Does it provide details of additional sources of support and information?	2.78 (0.89)	1.67	5
	Q8	Does it refer to areas of uncertainty?	2.56 (0.87)	1	4.67
**Domain 2** Quality of medication information	**Q9—Q15**		**13.86 (3.62)**	**9.66**	**22.34**
	Q9	Does it describe how each treatment works?	2.74 (1.34)	1	5
	Q10	Does it describe the benefits of each treatment?	1.24 (0.39)	1	2
	Q11	Does it describe the risks of each treatment?	1.66 (0.80)	1	3.67
	Q12	Does it describe what would happen if no treatment is used?	1.58 (0.90)	1	4.33
	Q13	Does it describe how the treatment choices affect overall quality of life?	1.62 (0.65)	1	4
	Q14	Is it clear that there may be more than one possible treatment choice?	1.53 (0.81)	1	3
	Q15	Does it provide support for shared decision-making?	3.51 (0.94)	1	4.33
**Domain 3**	**Q16**		**2.83 (0.76)**	**1.67**	**4.67**
Overall quality of information provided by the online platform for retail of prescription medicines	Q16	Based on the answers to all of the above questions, rate the overall quality of the publication as a source of information about treatment choices.	2.83 (0.76)	1.67	4.67
	**Total Score**		**40.48 (7.20)**	**30.33**	**56.67**

**Note:** Scores are presented as the mean of three independent evaluators using a 5-point Likert scale (1 = strong disagreement, 5 = strong agreement with the item’s quality statement). Higher scores indicate better perceived information quality. SD = standard deviation

The highest and the lowest mean item Likert scores of 34 online platforms across the 16 DISCERN items were 4.09 and 1.24, respectively. The mean subtotal Likert score of the Domain 1 for 34 online platforms (Q1–Q8) was 23.79 (lowest: 20.99, highest: 26.17), with four scores below the median value. Some online platforms presented outdated and invalid information on the homepages of the websites, which were scored low on information source disclosure (Q4), when the information was produced (Q5), availability of additional support or information services (Q7), statements of uncertainties (Q8). Many online platforms were in short of providing dosing instructions, potential drug-drug interactions, or a comprehensive list of side effects. The mean subtotal Likert score of the Domain 2 for 34 online platforms (Q9–Q15) was 13.86 (lowest: 10.65, highest: 16.00). Only Q15 (3.51) reached the median value. The key identified problems were insufficient information about mechanism of action (Q9), treatment benefits (Q10), treatment risks or adverse drug reactions (Q11), risk of discontinuing medication (Q12), impacts on the quality of life (Q13), and other treatment options (Q14). Notably, some online platforms provided insufficient descriptions of treatment risks (Q11) and failed to emphasize the importance of professional medical advice or the limitations of the online information (Q8). In addition, the readability of the information provided by some online platforms was poor, which was not consumer-friendly, and possibly lead to confusion. The mean Likert score of the Domain 3 for 34 online platforms (Q16) was 2.83 (lowest: 2.33, highest: 3.00). The mean overall Likert score for 34 online platforms was 40.48 (*SD* = 7.20; Table 1).

Among the 34 online platforms, two were rated as *Excellent* (5.88%), 13 were rated as *Good* (13/34, 38.24%), and one was rated as *Poor* (1/34, 2.94%) for the Domain 1 in the domain level categorical evaluation based on the above scores. 19 were rated as *Poor* (19/34, 55.88%) for the Domain 2. Two were rated as *Excellent* (2/34, 5.88%), six were *Good* (6/34, 17.65%), 25 were *Fair* (25/34, 73.53%) and one was *Poor* (1/34, 2.94%). and only one was rated as *Poor* (1/34, 2.94%) for the Domain 3 (Table 2).

**Table 2:**
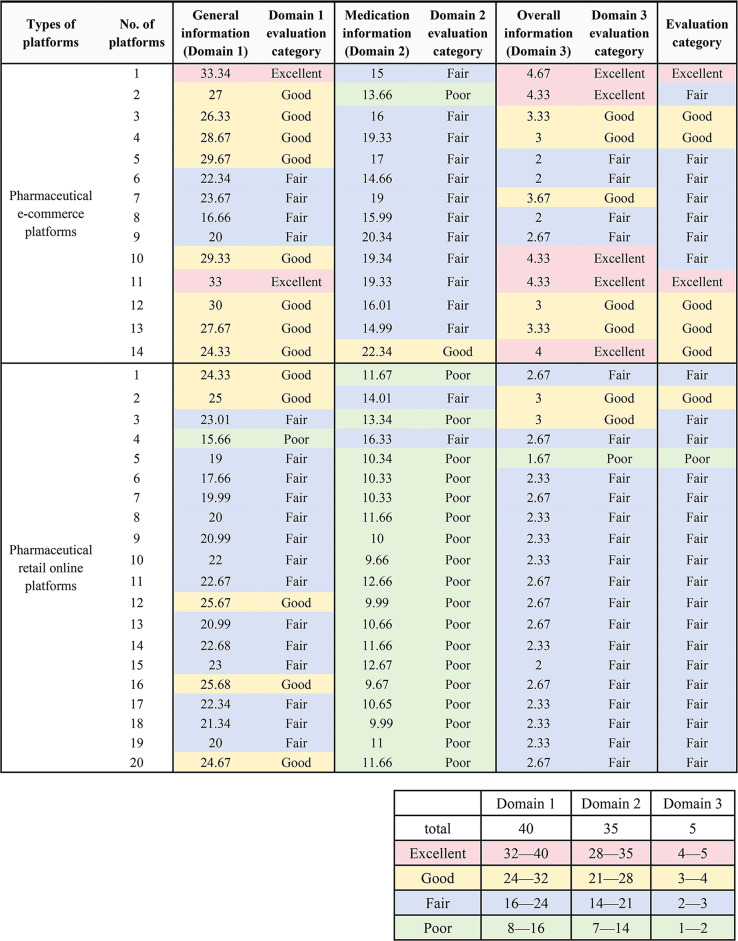
Domain-level and overall scoring results and categorical evaluation results for 34 online platforms

**Note:** Names of 34 online platforms providing retail service for prescription medicines were listed in Appendix 1

The Shapiro-Wilk test indicated a non-normal distribution of the scoring results between two types of online platforms (*W* = 0.923, $p = 0.02$). As presented in Fig. 2(b), the Wilcoxon rank-sum test result indicated that there were statistically significant differences of the mean Likert scores for 10 of the 16 items between two types of online platforms. That for the pharmaceutical e-commerce platforms were better than that for the pharmaceutical retail online platforms.

## Discussion

This study evaluated the quality of the information provided by the online platforms for retail of a common mental health prescription medicine in China. Less than 6% of the investigated online platforms performed excellently. Most were poor in compliance with the national Provisions for Supervision and Administration of Pharmaceutical Online Sales. Across all the investigated online platforms, the left-skewed distribution of the Likert scores of the 16 items implied poor performance in: when the information was produced (Q5), availability of additional support or information services (Q7), statements of uncertainties (Q8), treatment benefits (Q10), treatment risks or adverse drug reactions (Q11), risks of discontinuing medication (Q12), impacts on the quality of life (Q13), and other treatment options (Q14). Inadequate provision of information—particularly regarding treatment risks (Q11), alternative options (Q14), and the implications of non-treatment (Q12)—may mislead consumers. This could lead to inappropriate decisions regarding paroxetine use, including incorrect treatment selection, improper utilization, or medication errors, with potential serious safety implications. Our study found that the pharmaceutical e-commerce platforms performed better than the pharmaceutical retail online platforms in terms of providing comprehensive, accurate and reliable information for retail of prescription medicines. This advantage is attributed to the fact that, the former function as direct brand extensions, and usually provide uniform stringent control of the information provided for online retail of prescription medicines. They ensure data reliability through direct sourcing of information from the pharmaceutical manufacturers. Holding the third-party online platforms accountable will help compliance with any regulatory measures imposed on individual online retail stores. On the other hand, the latter act as intermediaries between the online and the online business, which often leads to inconsistencies in the quality and completeness of the information provided. This is often due to the integration of contents from multiple sources and a less centralized approach to oversight and quality assurance, and potentially associated with inconsistencies due to lack of rigorous content curation.

The medication instructions provided by the online platforms were found to be incomplete and insufficient. Critical gaps included unclear descriptions of how the treatment works (Q9), inadequate details about its benefits (Q10) and risks (Q11), and a lack of information on how treatment affects quality of life (Q13). Although the online platforms all provided patient information leaflet (PIL), the consumer experiences varied significantly. The PIL should have played an important role in making up for these deficiencies [54]. To secure transparency and ensure consumer protection, specific regulatory bodies in the EU and the USA have been designated to oversee the control and accuracy of PIL provided on the online pharmaceutical platforms. In the EU, pharmaceutical e-commerce platforms and individual online pharmacies must register with the European Medicines Agency and provide comprehensive documents to validate the legitimacy of their operations. Similarly, in Germany, the Federal Institute for Drugs and Medical Devices requires pharmacies authorised for mail-order and other online sales to prominently display the EU security logo on their websites. The U.S. Food and Drug Administration has taken regulatory actions to restrict the online sale of certain high-risk prescription drugs [55]. For example, through its BeSafeRx campaign, FDA warns consumers about counterfeit or unsafe online medicines and provides guidance on how to identify legitimate online pharmacies [56].

We also found that none of the online platforms implemented clear procedures for prescription validation and documentation, neither the scanned nor the electronic copies. The entire purchasing process also did not have professional assessments, recommendations, and alerts, including for those on treatment and allergy history, use and dosage, and adverse drug reactions. Although the online platforms set online questionnaires to inquire about the health conditions, allergies, and medical history of the patient, the consumer simulators could still get direct access to the webpage of medicines directly, bypassing the questionnaires through the default settings or the pre-selected negative answers without entering any specific information [57]. Such ‘no-prescription’ internet pharmacies pose significant risks to safe medication practices [58] and may be more likely to create exposure to medication errors [59]. Possible regulatory options could include a digital pharmacy accreditation system like in the United States, which facilitates electronic prescription identification and authentication between the online pharmacy and the electronic prescription systems of hospitals and physicians [60]. The Spine central prescription database in the UK plays a similar role. Additionally, the Quick Response Code of prescription and the long-term prescription program for contracted chronic patients in the UK provide convenience to patients and reduce illegal practices [61]. This study is the first comprehensive quality evaluation of the information provided by the online platforms for retail of prescription medications in China based on a scientific evaluation scale and with the perspectives of consumers. By extension, it constitutes a preliminary account of current insufficiencies. While paroxetine serves as a representative case for a widely used prescription medication with specific safety considerations, the extent to which these findings generalize to other therapeutic classes (e.g., antibiotics, cardiovascular drugs) or higher-risk medications requires further investigation. Information quality and compliance may vary based on a drug’s risk profile, public familiarity, and market dynamics.

To address these insufficiencies, a multi-faceted response is required, clearly delineating responsibilities between regulatory enforcement and platform self-regulation. The primary onus lies with regulatory authorities to mandate and enforce foundational safety standards. This should include the establishment of a compulsory digital pharmacy accreditation system, the enforcement of robust and verifiable prescription validation protocols to curtail non-prescription sales, and the setting of minimum mandatory requirements for the accuracy and comprehensiveness of medication information disclosed online. Concurrently, online platforms themselves must engage in proactive self-regulation and industry leadership beyond mere compliance. This entails voluntarily enhancing the clarity and accessibility of patient information materials, investing in integrated consumer education initiatives about medication safety, optimizing platform design and search functionality to prioritize reliable content, and developing elevated standards for pharmacist consultation services [62]. Ultimately, the synergy of stringent regulatory oversight and conscientious platform self-improvement will be pivotal in ensuring that the convenience of online pharmaceutical retail does not compromise, but actively promotes, information quality and patient safety.

### Limitations

This study has several limitations. First, the DISCERN instrument was originally developed to evaluate static health information websites and has not been widely validated for assessing dynamic e-commerce platforms retailing prescription medicines. To enhance its applicability, we formulated detailed, context-specific evaluation criteria by integrating key requirements from China’s Provisions for the Supervision and Administration of Pharmaceutical Online Sales. Nonetheless, the scoring retains an element of subjective judgment, despite our rigorous training and high inter-rater reliability measures. Second, the findings are based on a case study of a single medication, paroxetine. While it is a clinically relevant tracer drug, the quality of information and compliance may vary for other therapeutic classes, limiting immediate generalizability. Third, our sampling strategy focused on major B2C platforms and the apps of large-scale O2O retailers, potentially excluding smaller businesses whose practices might differ. Fourth, the cross-sectional assessment provides only a snapshot in time; platform content, features, and regulatory compliance are dynamic and may evolve rapidly. This timeframe also coincides with the early implementation phase of the 2022 Provisions, meaning some observed deficiencies might reflect transitional challenges rather than settled non-compliance. Finally, while our protocol aimed for consistency, we could not control for all potential external variabilities, such as differences in information presentation based on user geographic location or login status.

## Conclusions

Online retail of prescription medications is still in the early development stages in China. Using paroxetine as a case study, this investigation found that consumers faced challenges in obtaining comprehensive, accurate and reliable information from the online platforms for common mental health prescription medications. The quality of information provided by the online platforms for retail of prescription medicines is poor, and the compliance with prescription validation and authentication is problematic. These highlight the need for strengthening the supervision of the online platforms with novel approaches along with their rapid developments, and ensuring appropriate online information provided to the consumers.

## Electronic supplementary material

Below is the link to the electronic supplementary material.

Supplementary Material 1

## Data Availability

All data and materials relevant to the study are included in the article or uploaded as supplementary information.
