# Supplementary material for: Quality evaluation of online platforms information for retail of prescription medicines in China: an observational study of the case of paroxetine
Source: BMC Med Inform Decis Mak. 2026 Feb 21;26:93. doi: 10.1186/s12911-026-03386-4 (PMC13032684; doi:10.1186/s12911-026-03386-4)
Supplement: Supplementary file 1 — Supplementary Material 1 [file 12911_2026_3386_MOESM1_ESM.docx]

**Quality evaluation of online platforms information for retail of prescription medicines in China: an observational study of the case of paroxetine**

**Appendix 1** Target online platforms running retail services for prescription medications

| **Platform Classification** |  | **Name of platform** | **Website** |
| --- | --- | --- | --- |
| Pharmaceutical e-commerce platforms | 1 | JINDONG HEALTH | [https://pharma.jd.com/](https://pharma.jd.com/) |
|  | 2 | Ali Health | [https://maiyao.liangxinyao.com/](https://maiyao.liangxinyao.com/) |
|  | 3 | Fangzhou Jianke | [https://www.jianke.com/](https://www.jianke.com/) |
|  | 4 | Babaifang | [http://www.800pharm.com/](http://www.800pharm.com/) |
|  | 5 | KDL HEALTH | [https://www.baiji.com.cn/](https://www.baiji.com.cn/) |
|  | 6 | Changsheng Pharmacy | [http://www.360srcs.com/](http://www.360srcs.com/) |
|  | 7 | Yibang Medicine | [https://www.yaofangwang.com/](https://www.yaofangwang.com/) |
|  | 8 | Tuling | [https://www.315jiage.cn/](https://www.315jiage.cn/) |
|  | 9 | Saizilin Pharmacy | [http://m.360bzl.com/](http://m.360bzl.com/) |
|  | 10 | Yihao Pharmacy | [https://www.111.com.cn/](https://www.111.com.cn/) |
|  | 11 | Kangaido | [https://www.360kad.com/](https://www.360kad.com/) |
|  | 12 | Liang Jian Hao Yao Wang | [https://www.360lj.com/](https://www.360lj.com/) |
|  | 13 | Kangze Pharmacy | [http://www.173kz.com/](http://www.173kz.com/) |
|  | 14 | Dingdang Kuaiyao | [https://www.ddky.com/](https://www.ddky.com/) |
| Pharmaceutical retail online platforms | 1 | LBX Pharmacy Chain Joint Stock Company | LBX Pharmacy |
|  | 2 | Sinopharm Holding Guoda Pharmacy Co., Ltd. | Guoda Pharmacy |
|  | 3 | DaShenLin Pharmaceutical Group Co., Ltd. | DaShenLin Health |
|  | 4 | Yifeng Pharmacy Chain Co., Ltd. | Yifeng Pharmacy |
|  | 5 | Yixintang Pharmaceutical Co., Ltd. | Yixintang Pharmacy |
|  | 6 | Suyu Pingmin Chain Pharmacy Co., Ltd. | Suyu Pingmin Pharmacy |
|  | 7 | Liuzhou Guizhong Chain Pharmacy Co., Ltd. | Guizhong Pharmacy |
|  | 8 | Ruirentang Pharmaceutical Group Co., Ltd. | Ruirentang Health |
|  | 9 | Chongqing Hepin Chain Pharmacy Co., Ltd. | Hepin Pharmacy |
|  | 10 | Guangzhou Jianmin Pharmaceutical Chain Co., Ltd. | Guangzhou Jianmin |
|  | 11 | Shenzhen Nanbe Chain Pharmacy Co., Ltd. | Nanbei Pharmacy |
|  | 12 | Shandong Yanxi Tang Chain Pharmacy Co., Ltd. | Yanxi Tang |
|  | 13 | Chengdu Quan Yuan Tang Chain Pharmacy Co., Ltd. | Quan Yuan Tang Smart Pharmacy |
|  | 14 | Chongqing Wanhe Chain Pharmacy Co., Ltd. | Wanhe Yijia |
|  | 15 | Harbin Renmin Tongtai Chain Pharmacy Co., Ltd. | Renmin Tongtai Chain Pharmacy |
|  | 16 | Hunan Qianjin Chain Pharmacy Co., Ltd. | Qianjin Pharmacy |
|  | 17 | Shandong Lijian Chain Pharmacy Co., Ltd. | Lijian Youbaihui |
|  | 18 | Guangzhou Pharmacy Co., Ltd. | Guangzhou Medicine Pharmacy |
|  | 19 | Hunan Dajiaweikang Pharmaceutical Industry Co., Ltd. | Dajiaweikang Pharmacy |
|  | 20 | Zhongshan Zhongzhi Chain Pharmacy Co., Ltd. | Zhongzhi Pharmacy |

**Appendix 2** DISCERN scale

| **Sections** | **Items** | **Original questions** | **Evaluation criteria (1–5 points)** |
| --- | --- | --- | --- |
| Evaluation of prescription medicines online retail service information | Q1 | Are the aims clear? | Judge the quality of information presented on the homepage of the website of the pharmaceutical e-commerce platform (4 points for clear type of pharmaceutical e-commerce platform). Note: Only focus on the entrance to sell medicines; evaluation of the homepage of the website is not needed. |
|  | Q2 | Does it achieve its aims? | Determine whether the platform classifies commonly used medicines; this score should not exceed Q1. |
|  | Q3 | Is it relevant? | Determine whether antidepressant medicines can be found through the drug category information on the homepage. For example: ArkHealth - Commonly used medicines category 1 - Neurology 2 - Depression 3 - Paroxetine 5 (5 points for being able to find paroxetine through the website guidelines) |
|  | Q4 | Is it clear what sources of information were used to compile the publication (other than the author or producer)? | Determine whether a source is provided for the brand in question and assess its accuracy. For example: Jingdong Health homepage and Yifeng Pharmacy provides brand information (except for the pharmaceutical platform itself). 1 point for no source, 2 points for a source, and 3–5 points depending on the richness of the listed brands |
|  | Q5 | Is it clear when the information used or reported in the publication was produced? | Is it clear when the drug will be available? Or is there a prompt for new medicines? For example, Jingdong Health provides a display showing whether the drug is a new product of the season, prompting new products to score 3 points, and the remainder of cases compared to Jingdong Health scoring. |
|  | Q6 | Is it balanced and unbiased? | Conduct a drug search and judge according to the order of drug display. For example, if ‘paroxetine hydrochloride tablets’ is searched precisely, 5 points will be awarded if all recommended medicines are paroxetine hydrochloride and there is no evident brand ordering, 2-4 points will be awarded if all recommended medicines are paroxetine hydrochloride but there is an evident recommendation algorithm, and 1 point will be awarded if other medicines are also recommended. |
|  | Q7 | Does it provide details of additional sources of support and information? | Does it provide health information other than drug sales (e.g. health consultation and health Q&A)? 1 point for each item provided; maximum of 5 points |
|  | Q8 | Does it refer to areas of uncertainty? | The score for each item in Q7 determines whether there is variability in knowledge across sources. This score should not exceed Q7. If health advice on the site implies that patients choose to be treated in the same way or that a particular treatment has a 100% success rate, etc. no score is assigned. |
| Evaluation of medical information about paroxetine | Q9 | Does it describe how each treatment works? | Indications: This product is used for various types of depression, obsessive-compulsive neurosis, and also for the treatment of obsessive-compulsive disorder, panic disorder, and social anxiety disorder (1 point) Dosage: Oral, recommended to be taken daily at breakfast; swallow the tablets completely without chewing (1 point) Dosage: Differentiate dosage according to indications (1 point); differentiate according to the population of children/adults using the drug (1 point) Other clinical use guidelines or mechanism of action instructions (1 point) |
|  | Q10 | Does it describe the benefits of each treatment? | Depending on the evaluation, 1 point for listing (most pharmacies likely do not have efficacy statements) |
|  | Q11 | Does it describe the risks of each treatment? | 1, blood and lymphatic system; 2, immune system; 3, endocrine system; 4, metabolism and nutrition; 5, mental abnormalities; 6, nervous system; 7, eye; 8, cardiovascular system; 9, respiratory system, chest and mediastinum; 10, digestive system; 11, hepatobiliary system; 12, skin and subcutaneous tissue; 13, kidney and genitourinary system; 14, systemic and administration site reactions; 15, muscle skeletal system (1 point for each 3 items listed; the progression method is not used to score this item) |
|  | Q12 | Does it describe what would happen if no treatment is used? | Symptoms of paroxetine discontinuation. (1) Common: vertigo, sensory disturbance, sleep disturbance, anxiety, and headache (2) Uncommon: euphoria, nausea, tremors, confusion, sweating, and diarrhoea (3) As with several other psychotropic medicines, discontinuation of this product (especially when abruptly stopped) may result in symptoms such as vertigo, sensory disturbances (including sensory abnormalities, electroconvulsive sensations, and tinnitus), sleep disturbances (including intense dreams), euphoria or anxiety, nausea, headache, tremors, confusion, diarrhoea, and sweating. In most patients, these events were mild to moderate and self-limiting. No group of patients was found to be at higher risk for these symptoms, and hence it is recommended that paroxetine treatment should be tapered off if it is no longer needed. All three of the above listed scored 5 points, not all listed rated 2-4 points as appropriate, and not listed scored 1 point. |
|  | Q13 | Does it describe how the treatment choices affect overall quality of life? | 1, manic and bipolar disorders; 2, inability to sit still; 3, serotonin syndrome/antipsychotic malignant syndrome; 4, cardiac; 5, epilepsy; 6, glaucoma; 7, suicidal behaviour in children and adolescents (age < 18 years); 8, clinical deterioration and suicide risk associated with psychiatric disorders; 9, electroconvulsive therapy; 10, hyponatremia; 11, bleeding; 12, mono monoamine oxidase inhibitors; 13, monoamine oxidase inhibitors; 14, renal impairment/hepatic impairment; 15, driving/manipulating machines; 16, medication for pregnant and lactating women (1 point for each 3 listed; no progression for this score) |
|  | Q14 | Is it clear that there may be more than one possible treatment choice? | Recommendations for alternative options to be considered or further researched before deciding whether to select or reject a specific treatment option (including options not fully described in the publication) 1 point for a recommendation, with additional points awarded on a case-by-case basis depending on the presentation of the recommendation |
|  | Q15 | Does it provide support for shared decision-making? | Are patients prompted to seek advice on discussing treatment options with family, friends, doctors, or other health professionals? For example, is the doctor’s prescription truthfully charged?  There are 5 points for mandatory prescription uploading to sell medicines, 3 points for prompting to fill prescription information but not charging truthfully, and 1 point for no prescription prompting. |
| Overall Evaluation | Q16 | Based on the answers to all of the above questions, rate the overall quality of the publication as a source of information about treatment choices. | The platform has serious defects, 1; potential defects, 3; no evident defects, 5 |
